# Mr. Dunn on Extraordinary Abstinence

**Published:** 1813-08

**Authors:** John Dunn

**Affiliations:** Member of the Royal College of Surgeons, London. York


*To the Editors of the Medical and Physical Journal.*

GENTLEMEN,

IN consequence of the great attention which has been bestowed by the philosophical world to the impostor Ann Moore, together with the spirit of inquiry which still exists as to the possibility of the human animal subsisting without food, I have transmitted to you the following extracts from a paper in the Harleian Miscellany, which I thought at the least curious, and perhaps not without some degree of interest. The discourse was written by John Reynolds, and dedicated to Walter Needham, Doctor of Physic, and Member and Curator Elect to the Royal Society. It bears bears the date of 1669.* It is singular that the instance of reputed abstinence which he has related occurred also in Derbyshire. His exordium, consisting of a collection of similar instances, bears strong testimony of such occasional deviations from the course of nature; and I must confess, although at a loss to account for it, we are by no means to disregard such a mass of evidence, let the impositions have been ever so numerous. Many other facts less palpable to the community at large, and much less susceptible of proof, are believed, although equally inexplicable; and deceptions of this kind are so easily detected by the eye of the vulgar as well as that of the philosopher, we ought to be no less cautious in our rejection of what appears supernatural, than in giving it our implicit credence. Credulity and incredulity are alike the offsprings of unreflecting habits. Too great a pliability on the one side, and too much inflexibility on the other, are obstacles that will always interrupt the way to truth. That pen, however, as our author says, "certainly drops blasphemy, that dares to raze the sacred records; and that uncharitableness which presumes to write falsehood upon all human testimonies: they that assent to nothing not confirmed by authority, are unfit to converse in human societies; for how can I expect that anybody should believe me, whilst I myself will believe nobody? It is an argument of an empty brain, to presume to comprehend all things, and thereupon to reject those things from an existence in their world, that have not their science in its intelluals."

"Most certain it is, that the † learned Moses ‡ fasted forty days, and as many nights, whilst he abode in the burning mount; the great Elijah || went as long in the strength of a meal; and no less was the fast of the holy Jesus. § St. Austin ¶ reports, that, in his time, one survived forty days fasting.

---

* A Discourse upon prodigious Abstinence; occasioned by the twelvemonth's Fasting of Martha Taylor, the famed Derbyshire damsel: proving that, without any Miracle, the Texture of Human Bodies may be so altered, that Life may be long continued without the Supplies of Meat and Drink. With an Account of the Heart, and how far it is interested in the Business of Fermentation. By John Reynolds. Humbly offered to the Royal Society. London: Printed for Nevil Summons, at the Sign of the Three Crowns, near Holbourn Conduit, and for Dorman Newman, at the Surgeons Arms, in Little Britain. 1669. Quarto, containing 37 pages, besides the Title and Dedication.—*Harl. Miscell.* vol. iv. p. 43 et sequent.

† Acts vii. 22. ‡ Exod. xxx. 28. || 1 Kings xix. 8.

§ Matt. iv. 2. ¶ August. in Epist. 86. ad Cæsulanum.

But

But most strange is the story fathered on Nicephorus,* of three brethren affrighted by persecution into a cave, where they slept three hundred and seventy-three years, as was known by the coin they produced when they waked. The learned Fernelius † saith, he saw a pregnant woman that lived two months without meat or drink. Zacutus Lusitanus ‡ reports, that at Venice there lived a man that fasted forty days; another there forty-six days; and from Longius and Fontius (two considerable writers) another full three years; and that with just stature, good habit, free countenance, and youthful wit. The famous Sennertus || is copious in such stories: he relates from Sigismundus and Citesius, a person he saith worthy of credit, that the people of Leucomoria, inhabiting some mountains in Muscovy, do every year die, in a sort, (or rather sleep or freeze,) like frogs or swallows, on November 27, and so continue in that rigid state; the humor, distilling from their nostrils, is presently condensed by the ambient cold, much like to icicles, by which those potent pores are precluded, and the most endangered brain fortified against the fatal assaults of brumal extremities. The same Sennertus rehearses a story of a virgin at Padua, from Viguntia, professor there, who, anno 1598, was afflicted with a fever, then a tumor, then arthritic pains, and pains in the ventricle and whole abdomen; then with vomiting and nauseating of food, till at last she could take no food for two months; then, after another fit of vomiting, purging, and bleeding, she fasted eight months; and after a little use of food, she fasted two months more. And to be short, he stories it of three persons that fasted each two years, one three years, another four, one seven, another fifteen, another eighteen, and one twenty; yea one twenty-nine, another thirty, another thirty-six, and one forty years. Famous is the story, perhaps fiction, being poetical, of Epimenides,§ (whose words St. Paul is thought to cite in his Epistle to Titus,) whom some report to have slept seventeen years, some seventy-seven years together. But enough of story: those that are desirous to read more, are referred to Marcellus Donat. lib. iv.; de Med. Hist. Mirab. c. 12; Schenk, lib. iv.; Observ. Guaguinus, lib. iii.; Hist. Franc.

---

* Nicephor. lib. xiv. cap. 45.

† Fernel. lib. vi. Pathol. cap. 1.

‡ Zac. Lusit. de Medic. Princ. Hist. p. 914.

|| Sennert. Pract. lib. iii. par. 1. sect. 2. c. 3. De Longa Abstin, p. 383.

§ Vid. Sennert. ubi antea. Zac. Lusit. ubi antea. Plutarch in Sympos. et Lib. de Facie in Orb. Lunæ.

Petrarch, lib. iii.; de Mirabel, c. 22; Portius de Hist. Puellæ German.; Uspergensis in Chron.; Lentulus in Hist. Admir.; Apol. Berius, lib. de Vini Nutritione; Bozius, lib. xi. c. 4. de Signis Eccl.; Fulgorius, lib. i. c. 6; Lepæus, lib. ix.; Hist. Scot. Favorinus apud Gellium, lib. xvi. c. 3; and especially Licetus, who wrote a particular tract to solve the phenomena of this prodigy."

"But further to satisfy these incredulous persons, it is affirmed that some of these abstinents* have been watched by the most wakeful eyes and jealous ears, to detect their fraud, if guilty of any; as was that maid that refused all food, except only water, for three years, by Bucoldianus, with whom she abode for twelve days, at the command of Ferdinand the Emperor; so that Apollonia Schrejerana was taken by the senate of Bern, and put into the hospital of that town, and there watched till they were satisfied of the truth of her total abstinence."

Most of these cases are certainly too unnatural to attempt to refute, however gravely they may have been asserted. Useless, therefore, as the task would be to disprove what nobody would believe, as well as to combat with arguments the existence of what has been said to be seen, believed, and sworn to, it would be equally unjust to doubt the authenticity of the whole. The case which the author himself has related, bears strong testimony of the possibility of the human body subsisting under privations of food for a number of days, if we do not give credit for the full time he has represented. This abstinent, he says, "is one Martha Taylor, a young damsel born of mean parentage, inhabiting not far from Bakewell in Derbyshire; who, receiving a blow on the back from a miller, became a prisoner to her bed for several days; which being expired, she obtained some enlargement for a time, but by increasing distempers was quickly remanded to her bed-prison again; where continuing some time, she found, at last, a defect in her gula, and quickly after a dejection of appetite; so that, about the 22d of December, anno 1667, she began to abstain from all solid food, and so hath continued, (except something so small, at the seldom ebbings of her distemper, as is altogether inconsiderable,) till within a fortnight before the date hereof, which amounts to thirteen months and upwards; as also from all other sorts, both of meats and drinks, except now and then a few drops of the syrup of stewed prunes, water, and sugar, or the juice of a roasted raisin, &c. but these repasts are used so seldom, and in such very small quantities, as are prodigiously insuf-

* Sennert. ubi supra.

ficient ficient for sustentation; she evacuates nothing by urine or stool; she spits not, that I can hear of, but her lips are often dry, for which cause she takes water and sugar with a feather, or some other liquids; but the palms of her hands are often moist, her countenance fresh and lively, her voice clear and audible; in discourse she is free; her belly flapped to her back-bone, so that it may be felt through her intestines,* whence a great cavity is admitted from the cartilago ensiformis to the navel; and though her upper parts be less emaciated, (though much too,) yet her lower parts are very languid, and unapt for motion, and the skin thereof defiled with a dry pruriginous scurf, for which, of late, they have washed them with milk; she sleeps so sparingly, that once she continued five weeks waking. I hear nothing of any extraordinary previous sanctity,† though since her affliction, being confined to her bed, which lieth in a lower room by the fire-side, she hath learned to read; and being visited so plentifully by the curious from many parts, as also by the religious of all persuasions, she hath attained some knowledge in sacred mysteries, but nothing of enthusiasm, that she pretends unto. And, lest she should prove a cheat, she hath been diligently watched by physicians, surgeons, and other persons, (for at least a fortnight together,) by the appointment of the noble Earl of Devonshire, as is already published by Mr. Robins, B. of D., that is, ballad-maker of Derby; whose ballad, they say, doth much excel his book. Likewise several other persons, at other times, have been pleased to watch for their own satisfaction, who, detecting no fraud, have given the account above mentioned; which was, for the main, confirmed to me by a sophy, the renown of whose wisdom hath often made England to ring, who assured me that he had an exact account of her."

It was observed by Dr. Henderson, from Magn. Gabr. Block. that all examples of extraordinary fasting have been confined to the female sex. This is another confirmation of the remark. Men, however, under circumstances of necessity, have been enabled to endure severe privations, even under considerable bodily exertions. The crew of Bligh,‡ and the history of many other navigators, give full testimony

* This appears to have been the same with Ann Moore, in her last stage of abstinence.

† In the time of Reynolds and his predecessors, it was generally supposed that food was supplied these abstinents by angels or dæmons. To prove that this was not the case, he has particularly mentioned her being a person of no great sanctity.

‡ Vide Thornton's Medical Extracts.

of the powers that exist in mankind when their natural support has been materially reduced, and also totally taken away.

The principle of life in the torpid animals is certainly capable of being maintained by some mysterious laws quite independent of circulation, secretion, or digestion; for, according to the observation of Hunter,* a portion of food put into the stomach of one of these animals in their dormant state, will not be acted upon till the season when its customary functions are renewed, and yet it continues subject to the properties of living matter. It resists putrefaction, and possesses muscular power whenever called into action by its proper stimuli.

Two circumstances in the human frame, however, I should conceive incompatible with a state of abstinence, the appearance of which would always lead me immediately to suspect imposture, the growth of the animal, and a healthy state of it. The intimate sympathy of the stomach with every part of the body, must, in every case of derangement, particularly in cessation of action, produce its correspondent effects upon the system at large; and that an animal will grow when it has no matter but air to feed upon, is quite repugnant to common sense. Reynolds has given in this tract a long and curious account of the mode in which life is supported, which, according to the notions of the times, he supposed to depend upon maintaining a regular supply of food, to repair the necessary waste from the secretions; on the preservation of natural heat; upon fermentation continuing in the blood; upon the supply of vital spirits resulting from

* J. Hunter on the Animal Economy, p. 194, 5, &c.—In the hedge-hog, by the experiments of Mr. Jenner, he found that while the heat of the stomach was at 30°, it had neither the desire nor the power of digesting food. "Spallanzani also mentions the slowness of digestion in serpents, and quotes Bomare, (Dict. d'Histoire Nat.) who gives an account of a serpent at Martinico, in whose stomach a chicken had remained for three months without being completely digested, the feathers still adhering to the skin." Mr. Hunter doubts the truth of this in such a warm climate: but he says, that "at Belleisle, in the beginning of the winter 1761-2, I conveyed worms, and pieces of meat, down the throats of lizards, when they were going into winter quarters, keeping them afterwards in a cool place. On opening them at different periods, I always found the substances which I had introduced, and without any alteration; sometimes they were in the stomach; at other times they had passed into the intestine; and some of the lizards that were preserved alive, voided them toward the spring, but with very little alteration in their structure."—*Hunter's Observations on Animal Economy*, p. 195, 6.

food and fermentation; and upon sleep, which was conceived to arise from fumes ascending to the brain from ingested food.

Each of these circumstances he has treated upon separately, as far as with regard to this fasting woman. The opinions advanced are too ridiculous to mention. They appear, however, to have satisfied him as to the veracity and probability of these anomalies. Such is the danger of theorizing and endeavoring to account for every thing. Although Darwin* very forcibly remarks, that to theorize is to think, yet we are too liable to think wrong, more especially on what we know but little of: indeed, it is only taking up our time in idle speculations, when it might be better employed in the observation of the things themselves. It is seeking, too often, shadows for the substance, and at the best it tends to idleness. When men can account for things, they forbear further examination; and the cases themselves, which ought to be established by incontestible evidence, are then only supported by the chimeras of their narrator.

As I do not recollect any reference being made to this old and extraordinary production, I have therefore troubled you with a few extracts and remarks from it.

I have the honor to be, Gentlemen,
Your most obedient and often obliged Servant,
JOHN DUNN,
Member of the Royal College of Surgeons, London.

*York, July* 4, 1813.

P. S. When I was in the neighbourhood of Burton-upon-Trent, with the Royal Cumberland militia, I remember being informed of another very extraordinary circumstance, besides that of Ann Moore, by some of the men. They assured me they had been to see a person who possessed the faculty of sight only on a Sunday. They gave me a reference to the person, which I have lost; perhaps you may learn the particulars of this case, as far as regards its identity or fallacy, from some of your correspondents in that quarter.

---

*To the Editors of the Medical and Physical Journal.*

GENTLEMEN,

MR. GRANGER having thought proper to forestal the Rev. Leigh Richmond's intended statement of the second watch and subsequent exposure of the impostor Ann Moore, I trust I shall be allowed to offer a few brief

---

* Darwin's Zoonom. Preface, p. 2.

